# Reflective practice and its role in facilitating creative responses to dilemmas within clinical communication - a qualitative analysis

**DOI:** 10.1186/s12909-016-0823-x

**Published:** 2016-11-24

**Authors:** Gabriele Lutz, Gudrun Roling, Bettina Berger, Friedrich Edelhäuser, Christian Scheffer

**Affiliations:** 1 Integrated Curriculum for Anthroposophic Medicine (ICURAM), Chair for Medical Theory, Integrative and Anthroposophic Medicine, Department for Health, Faculty of Medicine, Witten / Herdecke University, Gerhard Kienle Weg 4, 58313 Herdecke, Germany; 2 Department of Psychosomatic Medicine, Gemeinschaftskrankenhaus Herdecke, Herdecke, Germany; 3 Chair for Medical Theory, Integrative and Anthroposophic Medicine, Department for Health, Faculty of Medicine, Witten / Herdecke University, Witten, Germany; 4 Department of Early Rehabilitation, Gemeinschaftskrankenhaus Herdecke, Herdecke, Germany; 5 Department of Internal Medicine, Clinical Education Ward for Integrative Medicine (CEWIM), Gemeinschaftskrankenhaus Herdecke, Herdecke, Germany

**Keywords:** Creativity, Communication, Medical education, Reflective practice

## Abstract

**Background:**

Good communication is a major factor in delivering high quality in care. Research indicates that current communication skills training alone might not sufficiently enable students to find context-specific creative solutions to individual complex personal and interpersonal challenges in the clinical context. This study explores medical students’ experiences with real communication dilemmas in a facilitated group setting. The aims were to gain a better understanding of whether and, if so, how reflective practice can enhance students’ ability to find creative individual solutions in difficult communication situations and to identify factors within the reflective setting that foster their creative competency.

**Methods:**

Thematic content analysis was used to perform a secondary analysis of semi-structured interview data from a qualitative evaluation of a group reflective practice training for final-year medical students. The categories that arose from the iterative deductive-inductive approach were analyzed in light of current scientific understandings of creativity.

**Results:**

Reflection on real difficult clinical communication situations appears to increase medical students’ ability to handle such situations creatively. Although group reflection on clinical dilemmas involving personal aspects can stir up emotions, participating students stated they had learned a cognitive process tool that enhanced their communicative competence in clinical practice. They also described changes in personal attitudes: they felt more able to persevere and to tolerate ambiguity, described themselves more open and self-efficient in such complex clinical communication situations and thus more motivated. Furthermore, they reported on factors that were essential in this process, such as reflection on current and real challenges, a group format with a trainer.

**Conclusions:**

Reflective practice providing a cognitive process tool and using real clinical challenges and trainer support in communication education may provide learners with the skills and attitudes to develop creativity in practice. Implementing reflection training in clinical communication education may increase students’ overall communicative competency.

## Background

The importance and value of creativity is well recognized in many fields of society, be it education [1], politics [2], management [3, 4], natural sciences [5] or mathematics [6]. However, when physicians are using a standard procedure or diagnostic approach, creativity or risk-taking is not called for. In complex clinical situations, with their individual biomedical, cultural, psychosocial and economic factors, easy and straightforward standard solutions are often not available, and creative solutions must be sought in clinical practice in general and also in communicating effectively.

But what is creativity in the context of communication? A creative product in general is defined as a product that is novel and useful [7]. The novel solution does not have to be novel for mankind, like an invention but can include developing products at least novel to the originator [8]. In the clinical setting, these definitions mean that, if straightforward ways of communication cannot be found to come to a common understanding, new individual approaches must be developed, ones that are useful and adequate for those involved.

The following clinical example illustrates what is meant by finding creative approaches: A patient was admitted with severe abdominal pain. Diagnostic testing did not reveal any organic cause, and the medical student thought the patient might be simulating. He tried to explain that there was nothing wrong in the diagnostic tests, but the patient felt misunderstood and was angry. Despite having done what he felt was right, the student felt puzzled and was dissatisfied because he did not know what to do and could not solve the dilemma. He did not yet have the ability to find new and useful (thus creative) ideas about how to proceed. How could this creative competency best be developed?

As a big improvement in medical education in recent decades, communication skills’ training has been incorporated into many medical curricula. Nevertheless research indicates that skills training alone might not sufficiently enable students to competently deal with context-specific individual personal and interpersonal challenges [9–11], residents do not feel well enough prepared for real clinical communication [12, 13], and communicative competency tends to decline unless sustained [14]. In many fields, be they arts, sports, surgery or driving a car, after acquiring the basic knowledge and skills, there is a period where these skills are practiced in many variations in reality over and over again under the tutelage of peers and superiors in order to develop the expertise to apply knowledge and skills creatively to different individual situations [15]. In contrast, real clinical communication experience is currently rarely reflected or supervised in medical education [14]. Thus Salmon [16] pleaded for emphasizing creativity in clinical training, arguing that it would improve the students’ ability to find original solutions to unique communication needs.

This process of finding creative paths in communicative situations resembles improvisation in music, theatre or dance and, as such, requires practice. For example, in both the clinical and the jazz settings, the backgrounds of the individuals concerned differ, and they are required in that very moment to generate something new, something that has not happened before and that harmonizes. It must relate closely to what the other individuals are producing and planning to produce. Similarly, in clinical care, methods such as reflective practice and supervision [26] are used to tailor communication to individual characteristics of both the patient and the clinician. They involve dialogical and experiential experiences, contextual variability and ambiguity, students’ reflections on their own creativity and effectiveness and encounters with patients and ‘real patient learning’ [16].

In reflective practice groups, communicative and professional competency is trained by taking individual clinical dilemmas and trying to come to new and useful (creative) solutions through deep reflection. There are different reflective formats, including Balint-groups [17], supervision groups [18], longitudinal reflection groups in preclinical and clinical settings [19] and clinical reflection training [20].

For the student scenario described above, this approach could mean, for example, that, during clinical reflection in the group, the medical student was able to reflect the situation more deeply. He could step into the patient’s shoes and develop new ideas about the patient’s possible perspectives on the situation and her anger, which didn’t occur to him before. At the same time, the student’s own cognitive or emotional limitations or biases could become clearer and be adapted to suit the situation. With these new ideas in mind, he was able to develop options as to how he could approach the patient and readdress and resolve the tense situation in a more sensitive way adjusted to his and the patient’s needs.

Developing educational interventions that foster creative communicative competency requires insight into how creativity is produced and enhanced in communication. To obtain a clearer understanding of creativity in communication, two general theoretical models of creativity are described. Rhodes [21] (see also Table 1) identified four essential factors involved whenever creativity is produced: (a) a definition of the product that results from the creative activity, (b) the cognitive processes involved in the creation of ideas, (c) the person who creates, and (d) the place factors (environment) that foster creativity [21]. This model is called the four p’s model: product, process, person and place. It is used as a cornerstone of creativity conceptualization [22], and there has been a call to implement it in all research on creativity [23].

**Table 1:** The Characteristics of the combined creativity framework

4 p’s	Characteristics
Product	A product is creative, when it is “new” and useful”
Process Preparation Incubation Illumination Elaboration	Problem finding: recognizing that a problem exists, finding gaps, inconsistencies, or flaws Preparatory phase: gathering and reactivating relevant information Divergent thinking: broad attention, or associative thinking: generating alternative ideas Ideas churn around below the threshold of consciousness Aha moment, insight occurs, often not one single moment Convergent thinking: Evaluating, refining, and developing one’s idea
Person	Willingness to overcome obstacles and perseverance, willingness to tolerate ambiguity, openness towards experiences and complexity, self-efficacy and increased motivation.
Place (creativity fostering environments)	Presence of challenge and autonomy, social interaction and support

For factor a), the definition of creative products as being new and useful is widely accepted [24, 25]. The process factors (b) describe the cognitive stages that lead to creative production. They are generally taken from Wallas’ 1926 description of four stages in the creative process [26]: preparation (becoming immersed into the problem), incubation (divergent thinking, broad attention), illumination (Aha-moment), and elaboration (convergent thinking, judgment whether the insight is valuable). Further authors have elaborated on this concept: The personality factors (c) include the willingness to overcome obstacles and perseverance, the willingness to tolerate ambiguity, openness to experience and complexity, self-efficacy [27–29], and intrinsic motivation [4]. Place or environmental factors (d) contributive to the creative process were identified by Amabile as presence of challenge, autonomy, social interaction and support [30].

It is in particular the four stages described by Wallas that are visible in clinical reflective practice processes. In the preparation stage of reflective practice, students are asked to look for unsolvable situations they encountered (problem finding) and to bring them into class so they can be used as learning examples rather than just regarding them as a nuisance. In the incubation stage of the creative process, divergent thinking is trained. Divergent thinking is defined as “thinking that moves away in diverging directions so as to involve a variety of aspects and which sometimes leads to novel ideas and solutions” [31]. In this stage, stepping back and looking for (reflecting on) cognitive and non-cognitive, conscious and preconscious material [6, 32, 33] supports awareness of all possible aspects involved in the situation, especially those that were initially not visible or not considered useful. The aha-moment (illumination), the moment where new ideas arise, is usually not one single moment but several insights interspersed with periods of incubation and elaboration [32]. In the elaboration stage, convergent thinking is more prominent, thinking that brings together information focused on solving a problem [34]. The ideas generated are worked on to determine which individual solution might fit best for the specific needs of the individuals in that particular setting. The combination of both frameworks is shown in Table 1.

This study aimed to gain a better understanding of whether a student’s ability to find creative individual solutions in difficult communication situations can be enhanced by reflective practice and, if so, which factors of the reflective practice setting are contributive to fostering this competency.

## Methods

This study is based on a re-analysis of data from semi-structured interviews that originally took place within research into the effectiveness of clinical reflective practice [20]. Since the results of the original study suggested that students had become more creative as a result of the reflective process, we used the collected data to investigate in detail the issue of creativity. Since we aimed to understand the subjective perspective on developing medical students’ attitudes and skills, we applied a qualitative design. The underlying paradigm is critical theory [35]. We analyzed the data using thematic content analysis and, using an iterative deductive-inductive process, analyzed the evolving categories in the light of the current scientific understandings of creativity mentioned above, applying the four p’s and four stage process model. The creativity concept suggests ‘directions along which to look without prescribing what to see’ [36].

### Setting

The original reflective practice groups took place on a Clinical Education Ward for Integrative Medicine (CEWIM) [37]. There, final year students are responsible for patient care, and participants were asked to reflect on personal and interpersonal dilemmas faced during their clinical work. Topics often addressed are how to cope with death, conflicts with peers, patients, how to react to unprofessional behavior, or negative feelings towards patients. The setting and the process stages used in the evaluated reflective practice format are described in more detail elsewhere [20].

### Sample

Thirty students were contacted and 18 agreed to be interviewed on their experiences. Some were interviewed directly after the rotation; some had already been in residency two to three years. Demographics are also described elsewhere [20].

### Data collection

The interviews were conducted between 2010 and 2011. Medical students were asked open questions (see also [20]) about their learning conditions, expectations, meaningful episodes and experiences with and helpful and hindering aspects of reflective training.

The questions in the interview guide were general and aimed to cover a broad range of experiences and to get a sense of the subjective view of a student’s development. At the time, we were not specifically concerned with the concept of creativity, so the questions did not specifically target the topic of creativity.

Current practice in Germany does not require ethical approval for studies of this kind [38]; nevertheless, the welfare and protection of the participants were guaranteed by respecting their rights, privacy, dignity, and sensitivities. Interviewers were all experienced in conducting qualitative interviews. All interviews were audio-recorded and transcribed verbatim. An English native speaker translated the German quotes in this manuscript.

### Data analysis

First the interviews were read by GL and GR (second author and nursing scientist) and paraphrased by both coders to get a feeling for the material regarding codes relating to creativity features. Then, the four p’s and the four process stages were used in a deductive way as sensitizing concepts. They provided preliminary starting categories for the detailed thematic content analysis described by Green and Thorogood [39]. This content analysis was carried out with the aid of MAXQDA, software for qualitative data analysis, 1989 – 2015, VERBI Software (Consult. Sozialforschung GmbH, Berlin, Germany).

Both the paper paraphrasing and the detailed coding were done by GL and GR independently, whereby they each worked on a different half of the interviews. Both assessors inductively developed a preliminary thematic framework of primary and secondary themes. The subsequent categories were iteratively analyzed in light of prior theory, and further categories and subcategories evolved. Discrepancies about categories were resolved through discussion. When adaptations were made, codes were given memos and the categories were refined iteratively, going back and forth between theory and the categories, leading to a more comprehensive picture of the development of creative competency in students.

In a final step, the author not involved in the initial process of coding and category building (CS) read the transcripts and independently deductively tested the other two authors’ analyses and conclusions. Again, mutual consent over disagreements was reached.

## Results

The interviews contained sufficient data on the main creativity characteristics product, process, person and place factors (see also Table 1) related to their experience with reflective practice to substantiate the analysis. Nevertheless, since we did not address the topic of creativity specifically, the results are sometimes extrapolations of several quotes. The individual quotes given below sometimes only reflect one particular aspect of a characteristic.

### Product

Students described communication situations where they could not find satisfying solutions, i.e. they lacked the creative competency to come to new and useful solutions in difficult clinical communication situations. They experienced reflective practice as helpful in developing such new and useful solutions to currently unsolvable situations.

*“Well, it was a difficult situation for me … and it was actually a real relief that … such a specific possible solution was developed, one that I could implement and that then also, at least for me, really brought a sense of finding inner peace in this insoluble situation.”*

### Process

Students experienced in reflective practice that identifying problems and addressing them in a reflective or creative way can help them to reach satisfying conclusions. They stated how working through complex and unsolved problems with a reflective tool helped them to develop ideas about the needs and limitations of the other people involved and, at the same time, about their own feelings. After understanding these needs and feelings, new and, for those involved, useful and suitable individual solutions could be found. Through repeated training of this reflection process, students felt they had learnt a process tool or skill they could use in difficult situations.

*“Basically developing tools to deal with difficult situations.”*

### Person

Students related that, through reflective practice, they had gained a clearer expectation of how difficult it is to change behavior in oneself and in others. They had learnt that there are unique situations in which perfect, straight-forward solutions cannot be reached but where one has to negotiate creatively the best possible solution according to what those involved are able to do within their given constraints. It was therefore easier for them to address and persevere when faced with communicative obstacles.

*“For me, the main benefit is really that this […] will give me the ability to continually somehow do my best when dealing with patients or that what I know, that it will need time to develop. But I can follow this ideal more relaxed.”*

Students reported that they, in comparison to others, could see ambiguity as something normal and felt more able to handle complex and ambivalent situations. Ambivalent feelings became more tolerable.

*“But also of course just to leave it as it is and accept that that is the situation. So, when there is tension or when something is unfinished that one doesn’t have to solve it straight away … not to expect that everything always runs smoothly. But that it is, in fact, possibly normal that well, that there are difficult encounters and that one can have different perspectives towards something and that they sometimes collide with each other, like that … yes.”*

Working on uncertain and, at first sight, unsolvable situations allowed students to be more open to experience and complexity in such circumstances. They considered themselves to be more open towards situations where they felt they had personal deficiencies, towards colleagues and patients who did not act as expected.

*“That I don’t so easily just stick the patient into a specific category; that is simply a difficult patient… But I would now say, will those people benefit from me trying, somehow in some way, well, from [me] having a sort of other internal flexibility and keeping on trying to [approach] this with a certain amount of impartiality.”*

Several students expressed that they could handle difficult professional and communicative problems better. Being able to synthesize their own ideals with the given imperfectness of reality seems to provide the students with a sense of self-efficacy.

*“Well, I think that these kinds of conversations allow you to turn an overwhelming situation into one where one feels competent again. They allow you to develop tools to deal with difficult issues… I personally perceive this as the difference between feeling powerless and overwhelmed and a certain competency that can be achieved over time.”*

Participants related that the sense of self-efficacy and the revised and more realistic expectations seemed to help in maintaining intrinsic motivation or one’s ideals and staying committed.

*“And it keeps you excited too. Because frustrating experiences don’t remain frustrating but can be turned around somehow.”*

### Place

Students also appreciated the combination of real challenge and reflection. The clinical experience brought with it a responsibility to face complex situations more autonomously. Two aspects were prominently described: First, in the clinical setting, students reported being so busy with fitting into the new situation and developing clinical thinking that, when things became complex, moral inconsistencies, conflicts with patients and team conflicts were overlooked or passively considered as a nuisance and not actively addressed. These issues could therefore not be solved and led to frustration. The fact that students were asked to talk about personal situations in reflective training was considered helpful as they became more aware of dilemmas that need a deeper reflective approach. And participants also related having learnt not to simply react in a straightforward manner but to identify when to step back and to switch into a different process approach to the situation.

*“I have taken away with me a sort of, so to speak a heightened sensitivity and awareness of what such a situation with so-called, or for oneself, difficult patients involves. Now I will always, even now in my tasks, be relatively quickly aware because I have been sensitized to the issue […] and there is always such an attempt, then of course a step […] to take a step behind your current, primary feelings and to have a slight distance.”*

The second useful factor in taking problems directly from clinical care was that by taking their own unsolved problems and finally solving them in their individually suitable way those problems became meaningful learning experiences.

*“So it was really based on us and on our problems. It really did directly affect all of us and so we could really directly make something out of it all.”*

Students also valued the social interaction in the group format because when different members of the group looked at a given situation, more perspectives could be gathered and in the discussion additional ideas appeared. Thus they felt that the group offered more creative options for solutions.

*“The others always have an external view of the situation and can bring this in, something that always really helped me personally, introducing new aspects and possible solutions.”*

Students felt that the whole setting including having a trainer who moderated the reflective process, the group and the presence of real challenge encouraged them to face and actively address difficult situations with patients, colleagues and superiors. At the same time, the training provided enough support and appreciation that they could feel self-efficacious and competent enough to take risks and dare to handle new steps autonomously, where previously they would have felt insecure.

*“That was very helpful. Yes. And we were quite enthusiastic about it […]. [I] had the impression […] we always left feeling like ‘You can do it, I believe in you’. That had quite an impact and was just really supportive.”*

## Discussion

Using the creativity framework including product, process (preparation, incubation, illumination and elaboration), person and place criteria helped to gain insight into the role of reflective practice in facilitating creative responses to complex communication dilemmas:

### Can communicative creativity be enhanced through reflective practice?

Medical students described that, in their training, they experienced many complex clinical communication situations where they did not have enough creative competency to find satisfying individual solutions. They stated that, through reflective practice, they found new and useful (thus creative) ways to handle these situations. Thus the product criterion for creativity was met.

Regarding the process, they stated that they had acquired a process tool or skill they could use in difficult individual communication situations. The repeated experience of applying the reflective process steps on so far unsolvable situations gave participants the feeling of having learnt and internalized a process skill that improved their ability to create individual solutions in complex communication dilemmas. As already described, the stages of this process tool, preparation, incubation, illumination and elaboration, are used in creativity training [40], and they match reflective practice formats as well. A recent study shows that explaining and training these cognitive process skills and heuristics improves the effect of training [41]. Thus training reflective/creative heuristics in reflective practice seems essential.

They also reported several changes in personal attitudes, which they attributed to the training. They felt more willing to overcome obstacles and to persevere in complicated professional situations, more tolerant towards ambiguity, more open to experience and complexity, more self-efficacious, and they could thus maintain their intrinsic motivation. These attributes are described as features of creative personalities.

Since the intervention described here was short, it is very questionable whether it led to real personality changes. It is more likely to have simply helped the students to use and develop pre-existing personal resources in a useful way so that they could be put to good use in the rigid medical setting. This effect has also been described in other creativity training [29]. In general, there is evidence that the ability to display creative skills can be enhanced by proper interventions [41]. There is also evidence that training in general [42] and reflective approaches including supervision [18] can improve the transfer of professional and communication skills to the workplace [15, 18, 20].

This study underlines that a reflective practice may provide learners with the skills to develop creativity in practice. They seem to have acquired the skill to use a creative process tool in situations that cannot be solved by standard approaches, and they seem to have developed personal attitudes that help in obtaining creative practice results. Thus these results match Rhodes product, process and person criteria for creativity, and they therefore show that reflective practice may, indeed, provide learners with the skills to develop creativity in complex professional and communicative situations at work.

### Which factors in reflective practice are essential to improve creative communicative skills and attitudes?

Since there are many different approaches to reflective practice, the question remains as to which place factors are essential in the training design for the success of the approach. In our study, besides providing a process tool, two further major factors were revealed as helpful in finding creative solutions: reflection of real challenges and the group setting with trainer.

Using real rather than simulated clinical challenges as learning situations carries certain advantages regarding the development of creative communicative competency. Because of the responsibility students bear and the complexity of real clinical situations compared to simulated situations, students work more autonomously and face greater challenges. Participants in our study reported that, to comply with the clinical requirements, they tended to overlook communication dilemmas and thus missed experiences they could have learnt from and became frustrated. To find the problems that would benefit from a creative approach, students have to first become aware of them. This step is known to need special attention in training [32, 33, 43].

Since addressing stressful and personally difficult situations can induce insecurity and anger, experiencing the gain of the upfront investment into reflection in the clinical context is essential [32, 33, 44]. Students can learn that a decision to step back, to develop upfront time to think in new ways, to go into divergent thinking to create alternative ideas and convergent thinking to come to new and useful solutions really can help them and their patients. Therefore developing individual solutions and learning how to apply them in context instead of just reflecting backwards should be part of reflective practice.

Current reflective practice in medical education does not appear to regularly include cognitive heuristics that go through the whole loop from identifying the problem in real life, actively addressing it, establishing related divergent and convergent thought processes, developing an (for the patient and the student) individual solution and enacting it to reevaluate the developed option. The provision of such heuristics might help improve medical students’ communicative competency, also in the long term. Therefore using real situations for reflection raises autonomy and responsibility and makes the challenge more difficult, but also meaningful, for students, as well as providing a whole loop for reflection including finding problems and trying out solutions to see whether they work. Challenge and autonomy were described as creativity enhancing place factors [30] and Table 1.

Furthermore, the group and the trainer as sources of more ideas, social interaction and support and encouragement have been identified in our study and in the literature as place factors that are conducive to developing creative products [30, 32].

There is further scientific evidence that real challenges, group format and support from a group and trainer are essential factors in creative training and reflective practice and that the ability to display creative skills can be enhanced by suitable interventions [15, 20, 40, 41, 45, 46].

All in all, finding creative ways to apply acquired knowledge and skills in unique complex situations and thus to arrive at new and useful solutions would appear to benefit from reflective practice that provides for training a cognitive creative process tool with all the creative process stages in a reflection of real challenges, a group format and an experienced trainer who facilitates the process. This reflective practice training may, in turn, lead to changes in attitudes that are favorable towards creativity. Students need to experience and train the arising solutions if expertise in the field is to be developed and performance at the workplace changed.

Our study shows that, by enhancing creativity, reflective practice could improve creative communicative competency at work, and it also describes process and place factors that seem to enhance personal creative competency. Of course, the more knowledge and skills are available, the better the creative product: it is not a question of skills OR creativity; both are needed.

### Implications

Supplementing communication skills training with creative reflective training in real clinical settings could improve communication and professional competency. This improvement could, in turn, lead to enhanced and sustained motivation. We suggest that reflective practice should be incorporated into the latter part of clinical education at medical schools. To implement this change in postgraduate training, health organizations need to be convinced that reflective training would be a valuable investment since it strengthens the professionalization and communicative competency of their staff and would raise the motivation of young physicians and improve the quality of care. The identified process and place factors could help in implementing useful and sustainable reflective training for both students and health educators, allowing them to teach reflective practice more effectively.

### Strengths and limitations

The strength of this study is that, to the best of our knowledge, it is the first to investigate whether, in clinical communication, creativity, as defined in the scientific literature, can be trained and, if so, which factors should be included in the training. The fact that, even without being directly asked about creativity, students report characteristic experiences that closely match creativity criteria underlines the notion that reflective group practice using experiences from real clinical challenges could enhance creative competency in communication.

Despite these strengths, there are also limitations to this study. First, since the students were not specifically asked about features of creativity, the results are not always clearly represented in the individual quotes. The topic of creativity is compounded within the whole context of the interviews. Second, we have so far only evaluated the perspective of students within one setting, and therefore the generalizability of our findings is limited. Third, the heterogeneity of the sample was possibly not large enough. Since we only had 30 participants at the time of the study and only 18 consented to take part in the interview, the group might have been too homogeneous for sufficient scientific rigor. There may be selection bias due to the special character of the medical school, the special format of CEWIM, and the fact that students who agreed to take part in the interviews may have been particularly motivated and reflective. We were unsuccessful in our attempts to engage other, less motivated students in these interviews.

Furthermore, in our study, only one negative outcome of the reflective practice intervention was mentioned: One student described that after the training she felt she had related things to the group that were too intimate. There may well be more of these negative outcomes. However, students who experienced them might not have talked about them or they might not have joined the groups. Additionally, only one trainer was involved in the provision of the training. Therefore it remains unclear how students would experience the intervention if facilitated by a larger trainer pool, and research on reflective group training with more diverse groups and different trainers is warranted. Whether the experienced improvements effectively translate into practice is not clear and need clarifying. More studies should be carried out to examine the changes in skills, attitudes, and personality we found with this qualitative study.

## Conclusions

Creativity competency in communication and finding new and useful ways to resolve difficult individual situations seems to be necessary and may benefit from training. Reflective group practice may enhance the creative and idiographic competency to apply knowledge and skills in difficult professional or communication situations so that they can be resolved in a new and useful way. Training cognitive processes and heuristics on how to come to solutions can provide cognitive skills and lead to changes in personal attitudes. These skills and attitudes seem to enhance creative communicative competency. Process and environmental factors were found to be helpful in finding such solutions. Therefore such training could serve as one missing link when transforming professional and communicative knowledge and skills into performance at the workplace.
